# Incorporating Covariates into Measures of Surrogate Paradox Risk

**DOI:** 10.3390/stats6010020

**Published:** 2023-02-17

**Authors:** Fatema Shafie Khorassani, Jeremy M. G. Taylor, Niko Kaciroti, Michael R. Elliott

**Affiliations:** Department of Biostatistics, School of Public Health, University of Michigan, Ann Arbor, MI 48109, USA

**Keywords:** surrogate markers, surrogate endpoints, meta-analysis, causal association, covariate information

## Abstract

Clinical trials often collect intermediate or surrogate endpoints other than their true endpoint of interest. It is important that the treatment effect on the surrogate endpoint accurately predicts the treatment effect on the true endpoint. There are settings in which the proposed surrogate endpoint is positively correlated with the true endpoint, but the treatment has opposite effects on the surrogate and true endpoints, a phenomenon labeled “surrogate paradox”. Covariate information may be useful in predicting an individual’s risk of surrogate paradox. In this work, we propose methods for incorporating covariates into measures of assessing the risk of surrogate paradox using the meta-analytic causal association framework. The measures calculate the probability that a treatment will have opposite effects on the surrogate and true endpoints and determine the size of a positive treatment effect on the surrogate endpoint that would reduce the risk of a negative treatment effect on the true endpoint as a function of covariates, allowing the effects of covariates on the surrogate and true endpoint to vary across trials.

## 1. Introduction

Clinical trials often collect intermediate, or surrogate, endpoints other than their true endpoint of interest. Surrogate endpoints are chosen because they occur more frequently, are easier to measure, or occur more proximally to the treatment time. The use of surrogate endpoints can result in a reduction in the required sample size for a trial, leading to shorter trial duration, as well as reduced costs of conducting clinical trials. A good surrogate endpoint is one that accurately reflects the effect of a given treatment on the true endpoint of interest while incurring lower cost or taking less time to measure. Some examples of surrogate endpoints include tumor progression as a surrogate endpoint for cancer-specific mortality, or CD4 counts in blood as a surrogate endpoint for AIDS mortality.

There exist several approaches for evaluating the strength of proposed surrogate endpoints. The first formalized approach for surrogate endpoint validation was presented by Prentice in 1989, who suggested that, among other criteria, a good surrogate should be highly correlated with the true endpoint [1]. He provided a method to test the surrogate by including it in a regression model of the true endpoint with the treatment and checking if it would eliminate the coefficient of the treatment association with the true endpoint of interest [1]. Later work pointed out that this approach does not allow for causal claims about surrogate efficacy since it ignores the potential of confounders between the surrogate endpoint and true endpoint. Confounding is possible despite randomization, since the surrogate endpoint is measured after treatment [2].

Since then, there have been several approaches proposed to evaluate surrogates in a causal inference framework when data are available on a single trial in which both outcomes are measured. These methods can be categorized into two major types: “causal effects” and “causal association” [2-4]. The causal effects paradigm uses the potential outcomes framework, which considers all the outcomes that would be potentially observed if the treatment and placebo were both applied to each subject (a combination of the observed outcomes and counterfactual outcomes if a subject were assigned to the opposite treatment that they actually received) [5]. Once the potential outcomes are defined, we consider both treatment and surrogate endpoints to be separately manipulable and create potential outcomes based on all possible combinations of potential outcomes [2]. This allows the estimation of the total effect of treatment as the sum of direct effects of the treatment on the true endpoint and indirect effects of the treatment that go through the surrogate endpoint. An ideal surrogate would capture the majority of the indirect effect of the treatment on the true outcome of interest, leaving little direct effect of the treatment. In the causal association framework, only the treatment and not the surrogate is considered manipulable. To account for the fact that the surrogate endpoint is measured after treatment, the causal association framework conditions on the joint counterfactual values of the surrogate endpoint under both the treatment and the control. Both the causal effects and causal association approaches use models that are not entirely identifiable, since we never completely observe the counterfactual distribution. There is an alternate causal association approach, presented by Buyse et al. in 2000, in the meta-analytic setting, where data are available on multiple trials of the same treatment and surrogate combination [6]. This approach leverages data from multiple randomized trials to assess the effectiveness of a surrogate endpoint, allowing all parameters to be identified from the observed data [6]. This is the setting we consider in this paper.

The goal of measuring the validity of a surrogate is to make sure that a surrogate endpoint accurately captures the effect of the treatment on the true endpoint of interest. There have been several examples of surrogate endpoints that are positively associated with both the treatment and the true endpoint of interest but have not accurately predicted the treatment effect on the true endpoint. One notable example is in the development of a drug to fight ventricular arrhythmias, which were considered to be a surrogate for cardiac-related deaths. The drug was found to lower ventricular arrhythmias, and ventricular arrhythmias were positively associated with cardiac deaths, leading to the approval of the drug in clinical trials. Subsequent follow-up trials found that the drug was associated with a significantly increased risk of cardiac death [7]. The phenomenon is labeled the “surrogate paradox” [8]. The surrogate paradox occurs when the treatment has beneficial effects on the surrogate outcome, and the surrogate outcome is positively associated with the true outcome, yet the overall effect of the treatment on the true outcome is negative, leading to incorrect conclusions that can be potentially dangerous to public health. It has been shown that testing the efficacy of a surrogate endpoint under either the causal association or causal effects framework is not enough to fully preclude the risk of observing the surrogate paradox [8]. There are several situations in which the surrogate paradox may be observed [9]. The first is when a direct effect between the treatment and the true outcome runs in the opposite direction of the indirect effect of the treatment through the surrogate. The second is when there is uncaptured confounding between the surrogate and true endpoints. The third is when the effect of the treatment on the surrogate and true endpoints are different on the individual level, meaning that the positive effect of the treatment is experienced on the surrogate endpoint for some patients and on the true endpoint for a different set of patients. In his paper, Vanderweele discusses means of assessing the risk of surrogate paradox and concludes that the meta-analytic approach [6] is the most effective, since it studies the efficacy of a surrogate measure over multiple trials. Elliott et al. proposed measures to assess the risk of surrogate paradox in the meta-analytic causal association framework [10].

Treatments may have different effects on different patient subpopulations, and there is the possibility that some subpopulations in a study may be at a different risks of experiencing the surrogate paradox. To consider this possibility, in this paper, we propose extensions to the measures of surrogate paradox risk proposed by Elliott et al. [10] that incorporate covariate information. Without considering covariate information when measuring the risk of surrogate paradox, there is the possibility that a new trial in a new population with different covariate distribution than past studies could expose those patients to a higher risk of surrogate paradox than what was expected. Incorporating covariate information may allow us to identify groups that are at particular risk of experiencing the surrogate paradox and help design future trials that make use of that surrogate. In the following sections, we describe the Buyse et al. meta-analytic causal association setting [11], the proposed surrogacy paradox risk measures from Elliott et al. [10], and then propose methods for incorporating covariate information.

## 2. Background

For surrogate marker $S_{ij}$ and outcome measure $T_{ij}$, where i=1,…,N indexes the trials, and $j=1,\ldots n_{i}$ indexes the subjects in the ith trial, Buyse et al. [6] considered the following distributions:

(1) $$S_{ij}=\alpha_{S}+\beta_{S}Z_{ij}+a_{S_{i}}+b_{S_{i}}Z_{ij}+\epsilon_{S_{ij}}$$

(2) $$T_{ij}=\alpha_{T}+\beta_{T}Z_{ij}+a_{T_{i}}+b_{T_{i}}Z_{ij}+\epsilon_{T_{ij}}$$

where $Z_{ij}\in 0,1$ is an indicator of treatment assignment, and

$$\left(\begin{array}{c} \epsilon_{S_{ij}}\\ \epsilon_{T_{ij}} \end{array}\right)∼N_{2}\left(\left(\begin{array}{c} 0\\ 0 \end{array}\right),\sigma =\left(\begin{array}{cc} \sigma_{ss} & \sigma_{st}\\ & \sigma_{tt} \end{array}\right)\right),$$

and random effects

$$\left(\begin{array}{c} a_{S_{i}}\\ a_{T_{i}}\\ b_{S_{i}}\\ b_{T_{i}} \end{array}\right)∼N_{4}\left(\left(\begin{array}{c} 0\\ 0\\ 0\\ 0 \end{array}\right),D=\left(\begin{array}{cccc} d_{ss} & d_{st} & d_{sa} & d_{sb}\\ & d_{tt} & d_{ta} & d_{tb}\\ & & d_{aa} & d_{ab}\\ & & & d_{bb} \end{array}\right)\right).$$

From this distribution, we can calculate the causal effect of a treatment Z on the surrogate marker in the ith trial as

$$\Delta_{S_{i}}=E(S_{ij}(1)-S_{ij}(0))\;\;=E(S_{ij}|Z_{ij}=1)-E(S_{ij}|Z_{ij}=0)\;\;=\alpha_{S}+\beta_{S}+a_{S_{i}}+b_{S_{i}}-(\alpha_{S}+a_{S_{i}})\;\;=\beta_{S}+b_{S_{i}}$$

Similarly, the causal effect of a treatment Z on the outcome measure in the ith trial is

$$\Delta_{T_{i}}=E(T_{ij}(1)-T_{ij}(0))\;\;=E(T_{ij}|Z_{ij}=1)-E(T_{ij}|Z_{ij}=0)\;\;=\alpha_{T}+\beta_{T}+a_{T_{i}}+b_{T_{i}}-(\alpha_{T}+a_{T_{i}})\;\;=\beta_{T}+b_{T_{i}}$$

Buyse et al. used the above distribution to suggest a trial-level measure of surrogate validity called $R_{trial}^{2}$ [6]. $R_{trial}^{2}$ is the proportion of variance explained by the trial-level random effects associated with the surrogate and is defined by

$$R_{trial}^{2}=\frac{V(T(1)-T(0))-V(T(1)-T(0)\;|\;a_{S_{i}},b_{S_{i}})}{V(T(1)-T(0))}\;\;=1-\frac{d_{bb}-(\;d_{sb}\;\;d_{ab}\;){\left(\begin{array}{cc} d_{ss} & d_{sa}\\ d_{sa} & d_{aa} \end{array}\right)}^{-1}\left(\begin{array}{c} d_{sb}\\ d_{ab} \end{array}\right)}{d_{bb}}\;\;=\frac{(\;d_{sb}\;\;d_{ab}\;){\left(\begin{array}{cc} d_{ss} & d_{sa}\\ d_{sa} & d_{aa} \end{array}\right)}^{-1}\left(\begin{array}{c} d_{sb}\\ d_{ab} \end{array}\right)}{d_{bb}}.$$

**Figure 1.**
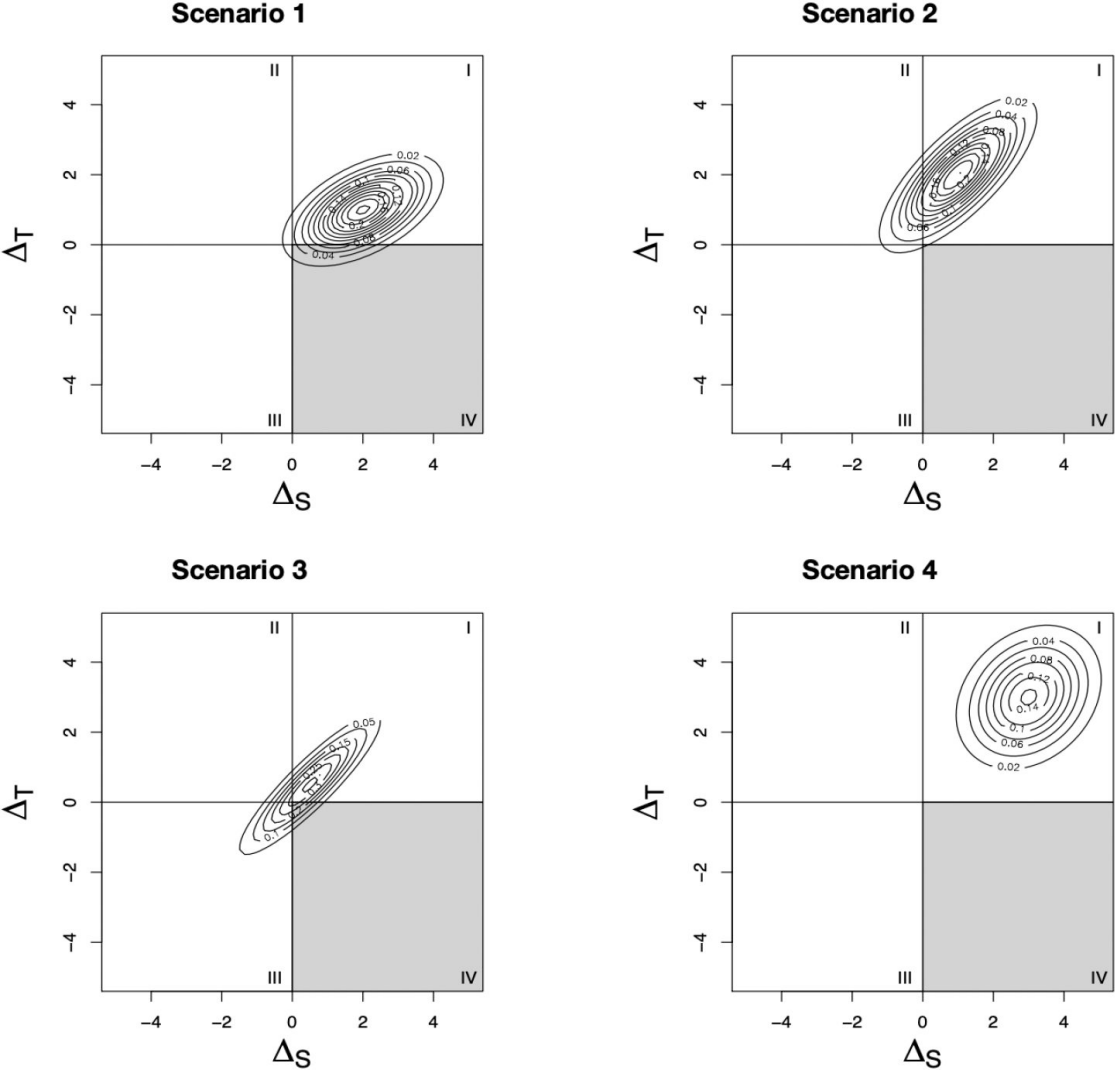
Joint distributions of the treatment effect on the surrogate and true outcome under four difference scenarios: (1) $\beta_{S}=2$, $\beta_{T}=1$, $d_{aa}=1$, $d_{ab}=0.375$, $d_{bb}=0.5$, $R_{trial}^{2}=0.358$, $\psi_{SP13}=0.918$, $\psi_{SP123}=0.931$; (2) $\beta_{S}=1$, $\beta_{T}=2$, $d_{aa}=1$, $d_{ab}=0.75$, $d_{bb}=1$, $R_{trial}^{2}=0.568$, $\psi_{SP13}=0.858$, $\psi_{SP123}=0.997$; (3) $\beta_{S}=0.5$, $\beta_{T}=0.5$, $d_{aa}=1$, $d_{ab}=0.9$, $d_{bb}=1$, $R_{trial}^{2}=0.822$, $\psi_{SP13}=0.874$, $\psi_{SP123}=0.937$; (4) $\beta_{S}=3$, $\beta_{T}=3$, $d_{aa}=1$, $d_{ab}=0.25$, $d_{bb}=1$, $R_{trial}^{2}=0.118$, $\psi_{SP13}=0.997$, $\psi_{SP123}=0.999$. $\psi_{SP13}$ is defined as the probability than an outcome and marker will have the same direction of treatment effects in a new trial and is introduced in Section 2.1. $\psi_{SP123}$ is defined as the probability of avoiding the dangerous surrogate paradox, or the situation in which the surrogate marker suggests a beneficial treatment effect but the outcome suggests a harmful treatment effect, and it is introduced in Section 2.2.

Elliott et al. use the joint distribution of $\Delta_{S_{i}}$ and $\Delta_{T_{i}}$. to develop several measures of surrogate paradox risk [10]. To do this, consider the contour plots of the joint distribution Figure 1. Throughout the paper, we assume, without loss of generality, that the qualitative effects of the treatment on the surrogate marker and true outcome are in the same direction, with positive effects beneficial and negative effects harmful. Each scenario shows the joint distribution of a different set of trials. Based on the location of the joint distribution on the Cartesian plane, we can infer the risk of surrogate paradox occurring. If the distribution falls mostly in the first or third quadrants, there is little risk of surrogate paradox, since $\Delta_{S}$ and $\Delta_{T}$ give the same qualitative conclusion. However, if the distribution falls in the second or fourth quadrants, the treatment effect on the surrogate and true outcomes are in opposite directions. By calculating the probabilities of the joint distribution falling in each quadrant, Elliot et al. present measures of the risk of surrogate paradox [10]. These measures are dependent on both the level of correlation between $\Delta_{S}$ and $\Delta_{T}$ and the size of the treatment effect on both outcomes. For example, in Scenario 1, although there is a strong correlation between the treatment effect on the surrogate and true outcomes, there is still some risk of surrogate paradox because of the relatively small treatment effect on the true outcome. In Scenario 2, there is some risk that the treatment effect on the surrogate outcome is negative, while the true treatment effect is positive; however, the increased true treatment effect size means that there is a lower risk of experiencing the more dangerous surrogate paradox (i.e., the treatment effect on the surrogate is positive while the true treatment effect is negative). In Scenario 3, despite the very strong correlation between the treatment effects on the two outcomes, there is some risk of surrogate paradox because of the low treatment effect sizes. Finally, in Scenario 4, there is low correlation between the two outcomes, but the risk of surrogate paradox is precluded because of the large treatment effect size on both outcomes. In the remainder of this section, we describe Elliott et al.’s measures of surrogate paradox risk using this joint distribution [10].

### 2.1. $\Psi_{SP13}$: Estimating the Probability That an Outcome and Marker Will Have the same Direction of Treatment Effects in a New Trial

The first surrogate paradox measure considers the probability that the N+1th trial will yield treatment effects on the marker and the outcome in the same direction. This probability is given by

$$\Psi_{SP13}=P(\Delta_{S,N+1}\times \Delta_{T,N+1}>0)=1-\Phi_{1}(0;\beta_{S},d_{aa})-\Phi_{1}(0;\beta_{T},d_{bb})+2\Phi_{2}\left(\left(\begin{array}{c} 0\\ 0 \end{array}\right),\left(\begin{array}{c} \beta_{S}\\ \beta_{T} \end{array}\right),\left(\begin{array}{cc} d_{aa} & d_{ab}\\ & d_{bb} \end{array}\right)\right)$$

where $\Phi_{k}(x;\Theta ,\Psi )$ is the cumulative distribution function of a k-variate normal distribution with mean Θ and variance Ψ. The subscript 13 in $\Psi_{SP13}$ refers to the first and third quadrants of the Cartesian plane, the region in which the marker gives a qualitatively correct prediction of the treatment effect.

### 2.2. $\Psi_{SP123}$: Estimating the Probability of Avoiding Dangerous Surrogate Paradox

A second measure of surrogacy paradox considers the particularly dangerous situation where the surrogate marker suggests a beneficial treatment effect but the treatment effect on the outcome measure is harmful. This probability is given by

$$\Psi_{SP123}=1-P(\Delta_{S,N+1}>0,\Delta_{T,N+1}<0)=1-\Phi_{1}(0;\beta_{T},d_{bb})+\Phi_{2}\left(\left(\begin{array}{c} 0\\ 0 \end{array}\right),\left(\begin{array}{c} \beta_{S}\\ \beta_{T} \end{array}\right),\left(\begin{array}{cc} d_{aa} & d_{ab}\\ & d_{bb} \end{array}\right)\right)$$

This measure estimates the probability that the N+1th trial lies outside of the fourth quadrant of the Cartesian plane (see Figure 1). It is the probability that a future trial will not result in a setting where the surrogate marker suggests the treatment will be helpful when, in fact, it is harmful.

### 2.3. ${\hat{\Psi }}_{SP13_{N}}$: Estimating the Probability That an Outcome and Marker Will Have the Same Direction of Treatment Effects in a New Trial When Partial Data Have Been Collected

The first two measures can be considered when drawing inferences about a future trial that has not yet collected data based on N historic trials that have already completed data collection. In practice, a trial may have already begun data collection and be interested in the risk of observing the surrogate paradox in their ongoing trial conditioning on the data from historic trials. In particular, they may have collected data on the surrogate outcome and no or very limited data on the true outcome of interest. We consider the situation where we have collected partial data for the Nth trial and want to estimate the measures of surrogate paradox risk in the ongoing trial conditioned on the previously collected data from the first N−1 trials.

Let ${ϒ}_{ij}\;=\;\left(\begin{array}{c} S_{ij}\\ T_{ij} \end{array}\right)$ constitute the surrogate marker and outcome for each subject, $M_{ij}=\left(\begin{array}{cccc} 1 & 0 & Z_{ij} & 0\\ 0 & 1 & 0 & Z_{ij} \end{array}\right)$ be the fixed effect matrix associated with the parameters $\mu =(\alpha_{S},\alpha_{T},\beta_{S},\beta_{T}{)}^{T}$, and let $W_{ij}=\left(\begin{array}{cccc} 1 & 0 & Z_{ij} & 0\\ 0 & 1 & 0 & Z_{ij} \end{array}\right)$ be the random effect matrix associated with $\gamma_{i}=(a_{S_{i}},a_{T_{i}},b_{S_{i}},b_{T_{i}}{)}^{T}$. Let ${ϒ}_{i}$, $M_{i}$, and $W_{i}$, represent the stacked elements of ${ϒ}_{ij}$, $M_{ij}$, and $W_{ij}$. Then, ${ϒ}_{N}$, $X_{N}$, and $W_{N}$ represent the stacked individual level data for each subject $\left(j=1,\ldots ,n_{N}\right)$ in the Nth trial (where $n_{N}$ is the total sample of the Nth trial so far) and $\gamma_{N}=(a_{S_{N}},a_{T_{N}},b_{S_{N}},b_{T_{N}}{)}^{T}$ represents the trial-level random effects.

The conditional distribution of $\gamma_{N}|{ϒ}_{N}$ can be found by considering the joint distribution of ${ϒ}_{N}\;∼\;N_{2n_{N}}(M_{N}\mu ,V_{N})$ and $\gamma_{N}\;∼\;N_{4}(0,D)$ and $cov({ϒ}_{N},\gamma_{N})\;=\;W_{N}D$ for $V_{N}=W_{N}DW_{N}^{T}+R$, with R representing a $2n_{N}\times 2n_{N}$ matrix with block diagonals of σ representing the individual level residual variance. Then, we have

$$\gamma_{N}|{ϒ}_{N}∼N_{4}({\tilde{\gamma }}_{N},{\tilde{D}}_{N})$$

where ${\tilde{\gamma }}_{N}=DW_{N}^{T}V_{N}^{-1}({ϒ}_{N}-X_{N}\mu )$ and ${\tilde{D}}_{N}=D-DW_{N}^{T}V_{N}^{-1}W_{N}D$. From here, the measure of surrogate paradox risk is given by

$${\hat{\Psi }}_{SP13_{N}}=1-\Phi_{1}(0;{\hat{\tilde{\beta }}}_{S_{N}},{\hat{\tilde{d}}}_{33_{N}})-\Phi_{1}(0;{\hat{\tilde{\beta }}}_{T_{N}},{\hat{\tilde{d}}}_{44_{N}})+2\Phi_{2}\left(\left(\begin{array}{c} 0\\ 0 \end{array}\right),\left(\begin{array}{c} {\hat{\tilde{\beta }}}_{S_{N}}\\ {\hat{\tilde{\beta }}}_{T_{N}} \end{array}\right),\left(\begin{array}{cc} {\hat{\tilde{d}}}_{33_{N}} & {\hat{\tilde{d}}}_{34_{N}}\\ & {\hat{\tilde{d}}}_{44_{N}} \end{array}\right)\right)$$

where ${\hat{\tilde{\beta }}}_{S_{N}}={\hat{\beta }}_{S}+{\hat{\tilde{b}}}_{S_{N}}$ for ${\hat{\beta }}_{S}$ corresponding to the third element of the maximum likelihood (ML) or reduced maximum likelihood (REML) estimate of μ and ${\hat{\tilde{b}}}_{S_{N}}$ corresponding to the third element of the ML/REML estimate of ${\tilde{\gamma }}_{N}$, ${\hat{\tilde{\beta }}}_{T_{N}}={\hat{\beta }}_{T}+{\hat{\tilde{b}}}_{T_{N}}$ for ${\hat{\beta }}_{T}$ corresponding to the fourth element of the ML/REML estimate of μ and ${\hat{\tilde{b}}}_{T_{N}}$ corresponding to the fourth element of the ML/REML estimate of ${\tilde{\gamma }}_{N}$, ${\hat{\tilde{d}}}_{kl_{N}}$ corresponding to the k, l element of the ML/REML estimator of ${\tilde{D}}_{N}$. Similarly, we can derive ${\hat{\Psi }}_{SP123_{N}}$.

This measure allows measurement of surrogate paradox risk after some data have been collected in the trial. This could be useful after the surrogate outcome has been collected on some of the patients, but there are not yet many (or any) measurements of the true endpoint that might occur later in the study. When $T_{Nj}$ is missing, ${ϒ}_{Nj}$ can be replaced with $S_{Nj}$ in the above calculations, while leaving the placeholder $X_{Nj}$ rows for the missing $T_{Nj}$.

### 2.4. s: Estimating the Size of the Beneficial Treatment Effect on the Marker Required to Preclude a Harmful Treatment Effect on the Outcome

In the fourth surrogate paradox measure, Elliott et al. consider the minimum observed beneficial treatment effect for a marker that can reduce the probability that the true treatment effect for the outcome is harmful. Let

$$O_{S_{i}}=\frac{\sum_{j}Z_{ij}S_{ij}}{\sum_{j}Z_{ij}}-\frac{\sum_{j}(1-Z_{ij})S_{ij}}{\sum_{j}(1-Z_{ij})}$$

represent the difference between the observed surrogate marker means under treatment and control. Note that for some value of s, $O_{S_{i}}$ will coincide with the true $\Delta_{S_{i}}$. Then, the joint distribution of the true treatment effect on the outcome and the observed treatment effect on the surrogate marker is given by

$$\left(\begin{array}{c} O_{S_{i}}\\ \Delta_{T_{i}} \end{array}\right)∼N_{2}\left(\left(\begin{array}{c} \beta_{S}\\ \beta_{T} \end{array}\right),\left(\begin{array}{cc} {\tilde{d}}_{aa} & d_{ab}\\ & d_{bb} \end{array}\right)\right)$$

where ${\tilde{d}}_{aa}=d_{aa}+\sigma_{ss}(1∕n_{1i}+1∕n_{0i})$, $n_{1i}=\sum_{j}Z_{ij}$ and $n_{0i}=\sum_{j}(1-Z_{ij})$. From here, they find that the distribution of the true treatment effect on the outcome $\Delta_{Ti}$ conditional on a given observed treatment effect $O_{Si}$ is

$$\Delta_{Ti}|O_{Si}=s∼N(\beta_{T}+d_{ab}∕{\tilde{d}}_{aa}(s-\beta_{S}),d_{bb}-d_{ab}^{2}∕{\tilde{d}}_{aa})$$

and

(3) $$P(\Delta_{Ti}<0|O_{Si}=s)=\Phi \left(\frac{-(\beta_{T}+d_{ab}∕{\tilde{d}}_{aa}(s-\beta_{S}))}{\sqrt{d_{bb}-d_{ab}^{2}∕{\tilde{d}}_{aa}}};0,1\right)$$

The authors propose two different ways to move forward from here. If data are collected to determine s, we can calculate the probability that the true effect in the outcome for the trial will be non-negative by replacing the parameters in (3) by their estimates from the data. Alternatively, we can determine the value of s that will ensure that the probability that $\Delta_{Ti}$ is negative is less than or equal to a preset level α:

$$s\geq \beta_{S}-\frac{{\tilde{d}}_{aa}}{d_{ab}}\left(\Phi (\alpha ;0,1{)}^{-1}\sqrt{\frac{{\tilde{d}}_{aa}d_{bb}-d_{ab}^{2}}{{\tilde{d}}_{aa}}}+\beta_{T}\right)$$

## 3. Incorporating Covariates

Treatments may have heterogeneous effects on surrogate and true endpoints in different patient populations, exposing some subpopulations to increased risk of surrogate paradox. Therefore, it is important that measuring risk of surrogate paradox allows consideration of patient level factors. To address this concern, a natural extension to Elliott et al. [10] is to incorporate covariate information by conditioning on a set of covariates and making the measures above (Sections 2.1-2.4) functions of covariates X. We can consider a situation where the surrogate and outcome measures depend on a set of covariates in addition to the treatment and extend (1) and (2) to incorporate covariates, where k=1,…,p indexes the number of covariates.


$$S_{ij}=\alpha_{S}+\beta_{S}Z_{ij}+\sum_{k=1}^{p}\gamma_{S_{k}}X_{ijk}+\sum_{k=1}^{p}\delta_{S_{k}}X_{ijk}Z_{ij}+a_{S_{i}}+b_{S_{i}}Z_{ij}+\sum_{k=1}^{p}c_{S_{i}}X_{ijk}+\sum_{k=1}^{p}d_{S_{i}}X_{ijk}Z_{ij}+\epsilon_{S_{ij}}\;T_{ij}=\alpha_{T}+\beta_{T}Z_{ij}+\sum_{k=1}^{p}\gamma_{T_{k}}X_{ijk}+\sum_{k=1}^{p}\delta_{T_{k}}X_{ijk}Z_{ij}+a_{T_{i}}+b_{T_{i}}Z_{ij}+\sum_{k=1}^{p}c_{T_{i}}X_{ijk}+\sum_{k=1}^{p}d_{T_{i}}X_{ijk}Z_{ij}+\epsilon_{T_{ij}}$$

This may be difficult to fit once p gets large and increases the number of random effects required. We consider two simplified scenarios that can be extended to a larger number of covariates if enough data are available:

- Scenario 1: The effects of covariates on surrogate and outcome are constant across trials (i.e., no random effects related to the covariates X).
- Scenario 2: The effects of covariates on surrogate and outcome are not constant across trials. In order to not overly complicate notation, we focus on the setting with only one scalar or binary covariate X (i.e., p=1, and all random effects related to the covariate X are included), but the approach can easily be extended to higher dimensions of covariates.

Although it is theoretically possible to consider a larger number of covariates, it is often not possible or computationally feasibly if it is expected that the effect of the covariates differs by study, since that would rapidly increase the size of the random effect variance matrix.

In the following two sections, we recreate the surrogate paradox measures from Elliott et al. under each of the above scenarios.

### 3.1. Scenario 1

Under scenario 1, we assume the effects of covariates on the surrogate and outcome measures are constant across trials:

$$S_{ij}=\alpha_{S}+\beta_{S}Z_{ij}+\sum_{k=1}^{p}\gamma_{S_{k}}X_{ijk}+\sum_{k=1}^{p}\delta_{S_{k}}X_{ijk}Z_{ij}+a_{S_{i}}+b_{S_{i}}Z_{ij}+\epsilon_{S_{ij}}\;T_{ij}=\alpha_{T}+\beta_{T}Z_{ij}+\sum_{k=1}^{p}\gamma_{T_{k}}X_{ijk}+\sum_{k=1}^{p}\delta_{T_{k}}X_{ijk}Z_{ij}+a_{T_{i}}+b_{T_{i}}Z_{ij}+\epsilon_{T_{ij}}$$

Then, we can choose a level $x_{k}$ for each $X_{k}$ in X and calculate the causal effect of a treatment Z on the surrogate marker among subjects with $X_{k}=x_{k}$ in the ith trial as

$$\Delta_{S_{i}}(x_{k})=E(S_{ij}(1|X_{ijk}=x_{k})-S_{ij}(0|X_{ijk}=x_{k}))\;\;=E(S_{ij}|Z_{ij}=1,X_{ijk}=x_{k})-E(S_{ij}|Z_{ij}=0,X_{ijk}=x_{k})\;\;=\alpha_{S}+\beta_{S}+\sum_{k=1}^{p}\gamma_{S_{k}}x_{k}+\sum_{k=1}^{p}\delta_{S_{k}}x_{k}+a_{S_{i}}+b_{S_{i}}-(\alpha_{S}+\sum_{k=1}^{p}\gamma_{S_{k}}x_{k}+a_{S_{i}})\;\;=\beta_{S}+\sum_{k=1}^{p}\delta_{S_{k}}x_{k}+b_{S_{i}}$$

Similarly, the causal effect of a treatment Z on the outcome measure among subjects with $X_{k}=x_{k}$ in the ith trial is

$$\Delta_{T_{i}}(x_{k})=E(T_{ij}(1|X_{ijk}=x_{k})-T_{ij}(0|X_{ijk}=x_{k}))\;\;=E(T_{ij}|Z_{ij}=1,X_{ijk}=x_{k})-E(T_{ij}|Z_{ij}=0,X_{ijk}=x_{k})\;\;=\alpha_{T}+\beta_{T}+\sum_{k=1}^{p}\gamma_{T_{k}}x_{k}+\sum_{k=1}^{p}\delta_{T_{k}}x_{k}+a_{T_{i}}+b_{T_{i}}-(\alpha_{T}+\sum_{k=1}^{p}\gamma_{T_{k}}x_{k}+a_{T_{i}})\;\;=\beta_{T}+\sum_{k=1}^{p}\delta_{T_{k}}x_{k}+b_{T_{i}}$$

Thus, $\Delta_{S_{i}}$ and $\Delta_{T_{i}}$ have the joint distribution:

$$\left(\begin{array}{c} \Delta_{S_{i}}\\ \Delta_{T_{i}} \end{array}\right)∼N_{2}\left(\left(\begin{array}{c} \beta_{S}+\sum_{k=1}^{p}\delta_{S_{k}}x_{k}\\ \beta_{T}+\sum_{k=1}^{p}\delta_{T_{k}}x_{k} \end{array}\right),\left(\begin{array}{cc} d_{aa} & d_{ab}\\ & d_{bb} \end{array}\right)\right)$$

**Figure 2.**
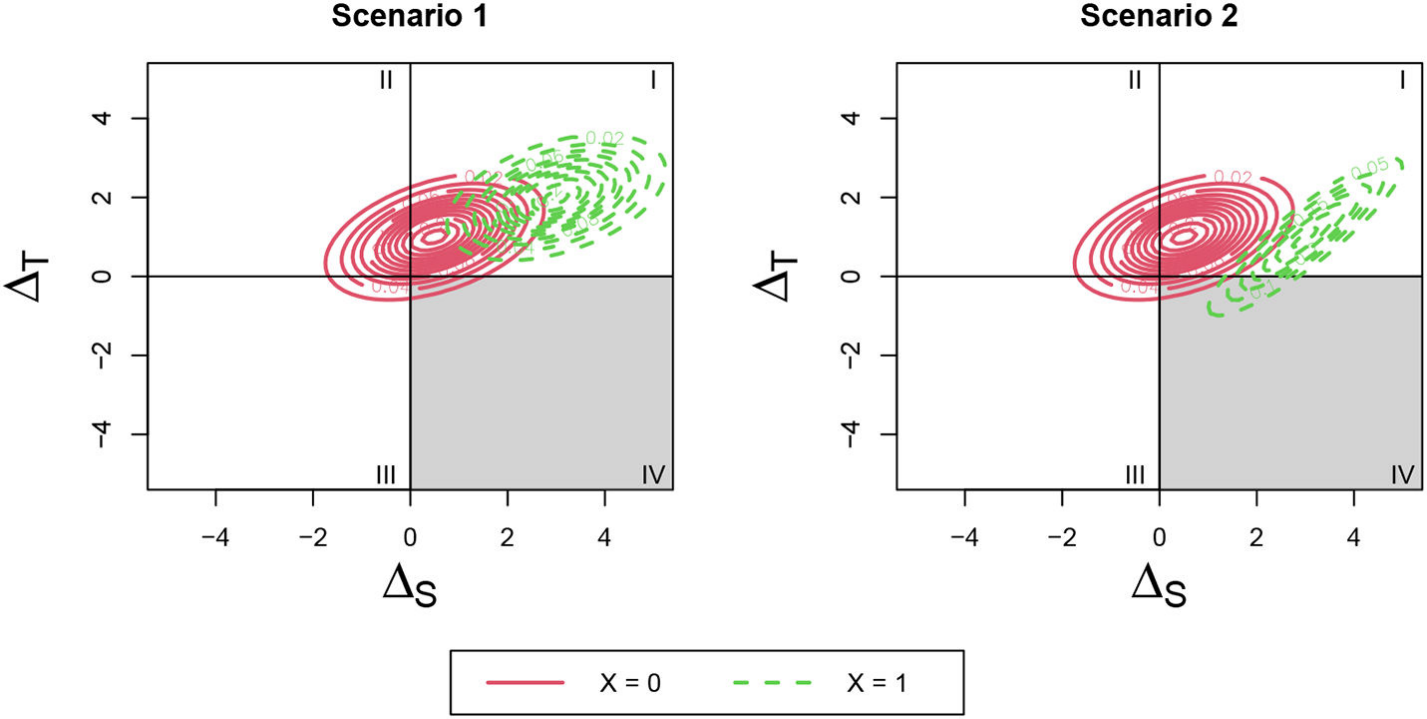
Changes to the joint distribution of $\Delta_{S}$ and $\Delta_{T}$ dependent on X: (1) Scenario 1: The effects of a binary covariate X on surrogate and outcome is constant across trials, resulting in a mean shift of the overall distribution for different levels of X. (2) Scenario 2: The effects of a binary covariate X on surrogate and outcome differs across trials, resulting in both a mean shift and variance change for different levels of X.

This distribution consists of a mean shift from the non-covariate-adjusted distribution. The variance remains the same as the original, no-subgroup distribution. To visualize this, refer to Scenario 1 in Figure 2. The risk of surrogate paradox may be different in the two groups and can be identified by calculating the differing probabilities of falling into each quadrant for the different covariate levels. The change in risk occurs from a mean shift of the overall joint distribution (the variance of the joint distribution for the two covariate levels remains the same).

#### 3.1.1. Scenario 1: $\Psi_{SP13}(x)$

Using the new joint distribution, the probability that the N+1th trial will yield treatment effects on the marker and outcome in the same direction is given by

(4) $$\Psi_{SP13}(x)=1-\Phi_{1}(0;\beta_{S}+\sum_{k=1}^{p}\delta_{S_{k}}x_{k},d_{aa})-\Phi_{1}(0;\beta_{T}+\sum_{k=1}^{p}\delta_{T_{k}}x_{k},d_{bb})+\;\;2\Phi_{2}\left(\left(\begin{array}{c} 0\\ 0 \end{array}\right),\left(\begin{array}{c} \beta_{S}+\sum_{k=1}^{p}\delta_{S_{k}}x_{k}\\ \beta_{T}+\sum_{k=1}^{p}\delta_{T_{k}}x_{k} \end{array}\right),\left(\begin{array}{cc} d_{aa} & d_{ab}\\ & d_{bb} \end{array}\right)\right)$$

where $\Phi_{k}(x;\Theta ,\Psi )$ is the cumulative distribution function of a k-variate normal distribution with mean Θ and variance Ψ.

#### 3.1.2. Scenario 1: $\Psi_{SP123}(x)$

Under the new joint distribution, the probability that the treatment effects for the outcome will be harmful given that the treatment effect on the marker is beneficial is given by

$$\Psi_{SP123}(x)=1-\Phi_{1}(0;\beta_{T}+\sum_{k=1}^{p}\delta_{T_{k}}x_{k},d_{bb})+\Phi_{2}\left(\left(\begin{array}{c} 0\\ 0 \end{array}\right),\left(\begin{array}{c} \beta_{S}+\sum_{k=1}^{p}\delta_{S_{k}}x_{k}\\ \beta_{T}+\sum_{k=1}^{p}\delta_{T_{k}}x_{k} \end{array}\right),\left(\begin{array}{cc} d_{aa} & d_{ab}\\ & d_{bb} \end{array}\right)\right)$$

This measure estimates the probability that a future trial will not result in a setting where the surrogate marker suggests the treatment will be helpful when it is, in fact, harmful.

#### 3.1.3. Scenario 1: ${\hat{\Psi }}_{SP13_{N}}(x)$

For this section, we consider the simplest case of one covariate for illustrative purposes. This can easily be extended to multiple covariates by extending the $X_{N}$ and $W_{N}$ matrices and the μ and $\gamma_{i}$ vectors. Let ${ϒ}_{ij}=\left(\begin{array}{c} S_{ij}\\ T_{ij} \end{array}\right)$ constitute the surrogate marker and outcome for each subject,

$$M_{ij}=\left(\begin{array}{cccccccc} 1 & 0 & Z_{ij} & 0 & x & 0 & xZ_{ij} & 0\\ 0 & 1 & 0 & Z_{ij} & 0 & x & 0 & xZ_{ij} \end{array}\right)$$

be the fixed effect matrix associated with the parameters $\mu =(\alpha_{S},\alpha_{T},\beta_{S},\beta_{T},\gamma_{S},\gamma_{T},\delta_{S},\delta_{T}{)}^{T}$, and let $W_{ij}\;=\;\left(\begin{array}{cccc} 1 & 0 & Z_{ij} & 0\\ 0 & 1 & 0 & Z_{ij} \end{array}\right)$ be the random effect matrix associated with $\gamma_{i}\;=\;(a_{S_{i}},a_{T_{i}},b_{S_{i}},b_{T_{i}}{)}^{T}$. Let $M_{i}$, ${ϒ}_{i}$, and $W_{i}$, represent the stacked elements of $M_{ij}$, ${ϒ}_{ij}$, and $W_{ij}$.

Consider the vector of random effects $\gamma_{N}\;=\;(a_{S_{N}},a_{T_{N}},b_{S_{N}},b_{T_{N}}{)}^{T}$, then the conditional distribution of $\gamma_{N}|{ϒ}_{N}$ can be found by considering the joint distribution of ${ϒ}_{N}∼N_{2n_{N}}(M_{N}\mu ,V_{N})$ and $\gamma_{N}∼N_{4}(0,D)$ and $cov({ϒ}_{N},\gamma_{N})\;=\;W_{N}D$ for $V_{N}=W_{N}DW_{N}^{T}+R$, with R representing a $2n_{N}\times 2n_{N}$ matrix with block diagonals of σ as before.

$$\gamma_{N}|{ϒ}_{N}∼N_{4}({\tilde{\gamma }}_{N},{\tilde{D}}_{N})$$

where ${\tilde{\gamma }}_{N}=DW_{N}^{T}V_{N}^{-1}({ϒ}_{N}-X_{N}\mu )$ and ${\tilde{D}}_{N}=D-DW_{N}^{T}V_{N}^{-1}W_{N}D$. From here, the measure of surrogate paradox risk is given by

$${\hat{\Psi }}_{SP13_{N}}(x)=1-\Phi_{1}(0;{\hat{\tilde{\beta }}}_{S_{N}}+{\hat{\tilde{\delta }}}_{S_{N}}x,{\hat{\tilde{d}}}_{33_{N}})-\Phi_{1}(0;{\hat{\tilde{\beta }}}_{T_{N}}+{\hat{\tilde{\delta }}}_{T_{N}}x,{\hat{\tilde{d}}}_{44_{N}})\;\;+2\Phi_{2}\left(\left(\begin{array}{c} 0\\ 0 \end{array}\right),\left(\begin{array}{c} {\hat{\tilde{\beta }}}_{S_{N}}+{\hat{\tilde{\delta }}}_{S_{N}}x\\ {\hat{\tilde{\beta }}}_{T_{N}}+{\hat{\tilde{\delta }}}_{T_{N}}x \end{array}\right),\left(\begin{array}{cc} {\hat{\tilde{d}}}_{33_{N}} & {\hat{\tilde{d}}}_{34_{N}}\\ & {\hat{\tilde{d}}}_{44_{N}} \end{array}\right)\right)$$

where ${\hat{\tilde{\beta }}}_{S_{N}}={\hat{\beta }}_{S}+{\hat{\delta }}_{S}+{\hat{\tilde{b}}}_{S_{N}}$ for ${\hat{\beta }}_{S}$ and ${\hat{\delta }}_{S}$ corresponding to the third and seventh elements of the estimate of μ and ${\hat{\tilde{b}}}_{S_{N}}$ corresponding to the third element of the estimate of ${\tilde{\gamma }}_{N}$, ${\hat{\tilde{\beta }}}_{T_{N}}={\hat{\beta }}_{T}+{\hat{\delta }}_{T}+{\hat{\tilde{b}}}_{T_{N}}$ for ${\hat{\beta }}_{T}$ and ${\hat{\delta }}_{T}$ corresponding to the fourth and eighth element of the estimate of μ and ${\hat{\tilde{b}}}_{T_{N}}$ corresponding to the fourth element of the estimate of ${\tilde{\gamma }}_{N}$, ${\hat{\tilde{d}}}_{kl_{N}}$ corresponding to the k, l element of the estimator of ${\tilde{D}}_{N}$. Similarly, we can derive ${\hat{\Psi }}_{SP123_{N}}(x)$.

#### 3.1.4. Scenario 1: s Value

In the fourth surrogate paradox measure, Elliott et al. consider the minimum observed beneficial treatment effect for a marker that can reduce the probability that the true treatment effect for the outcome is harmful [10]. When considering covariate subgroups, we can compute $O_{S_{i}}$ for each covariate level and call it $O_{S_{i}}(x)$:

$$O_{S_{i}}(x)=\frac{\sum_{j:X_{ij}=x}Z_{ij}S_{ij}}{\sum_{j:X_{ij}=x}Z_{ij}}-\frac{\sum_{j:X_{ij}=x}(1-Z_{ij})S_{ij}}{\sum_{j:X_{ij}=x}(1-Z_{ij})}$$

$O_{S_{i}}(x)$ represents the difference between the observed surrogate marker means under treatment and control within a fixed level of X. Then, the joint distribution of the true treatment effect on the outcome and the observed treatment effect on the surrogate marker is given by

$$\left(\begin{array}{c} O_{S_{i}}(x)\\ \Delta_{T_{i}}(x) \end{array}\right)∼N_{2}\left(\left(\begin{array}{c} \beta_{S}+\delta_{S}\;x\\ \beta_{T}+\delta_{T}\;x \end{array}\right),\left(\begin{array}{cc} {\tilde{d}}_{aa} & d_{ab}\\ & d_{bb} \end{array}\right)\right)$$

where ${\tilde{d}}_{aa}=d_{aa}+\sigma_{ss}(1∕n_{1ix}+1∕n_{0ix})$, $n_{1ix}=\sum_{j:X_{ij}=x}Z_{ij}$, and $n_{0ix}=\sum_{j:X_{ij}=x}(1-Z_{ij})$. So, the distribution of the true treatment effect on the outcome $\Delta_{Ti}(x)$ conditional on a given observed treatment effect $O_{Si}(x)$ within the group having X=x is

$$\Delta_{Ti}|O_{Si}(x)=s∼N(\beta_{T}+\delta_{T}x+d_{ab}∕{\tilde{d}}_{aa}(s-(\beta_{S}+\delta_{S}x)),d_{bb}-d_{ab}^{2}∕{\tilde{d}}_{aa})$$

and

$$P(\Delta_{Ti}<0|O_{Si}=s,X=x)=\Phi \left(\frac{-(\beta_{T}+\delta_{T}x+d_{ab}∕{\tilde{d}}_{aa}(s-(\beta_{S}+\delta_{S}x)))}{\sqrt{d_{bb}-d_{ab}^{2}∕{\tilde{d}}_{aa}}};0,1\right)$$

The value of s that will ensure that the probability that $\Delta_{Ti}(x)$ is negative is less than or equal to a preset level α:

$$s\geq \beta_{S}+\delta_{S}x-\frac{{\tilde{d}}_{aa}}{d_{ab}}\left(\Phi (\alpha ;0,1{)}^{-1}\sqrt{\frac{{\tilde{d}}_{aa}d_{bb}-d_{ab}^{2}}{{\tilde{d}}_{aa}}}+\beta_{T}+\delta_{T}x\right)$$

### 3.2. Scenario 2

Under scenario 2, we assume the effects of the covariates on the surrogate and outcome are not constant across trials. For simplicity, we consider only one scalar or binary covariate X:

$$S_{ij}=\alpha_{S}+\beta_{S}Z_{ij}+\gamma_{S}X_{ij}+\delta_{S}X_{ij}Z_{ij}+a_{S_{i}}+b_{S_{i}}Z_{ij}+c_{S_{i}}X_{ij}+d_{S_{i}}X_{ij}Z_{ij}+\epsilon_{S_{ij}}\;T_{ij}=\alpha_{T}+\beta_{T}Z_{ij}+\gamma_{T}X_{ij}+\delta_{T}X_{ij}Z_{ij}+a_{T_{i}}+b_{T_{i}}Z_{ij}+c_{T_{i}}X_{ij}+d_{T_{i}}X_{ij}Z_{ij}+\epsilon_{T_{ij}}$$

where

$$\;\left(\begin{array}{c} \epsilon_{{}_{S_{ij}}}\\ \epsilon_{{}_{T_{ij}}} \end{array}\right)∼N_{2}\left(\left(\begin{array}{c} 0\\ 0 \end{array}\right),\sigma =\left(\begin{array}{cc} \sigma_{ss} & \sigma_{st}\\ & \sigma_{tt} \end{array}\right)\right)\;\left(\begin{array}{c} a_{S_{i}}\\ a_{T_{i}}\\ b_{S_{i}}\\ b_{T_{i}}\\ c_{S_{i}}\\ c_{T_{i}}\\ d_{S_{i}}\\ d_{T_{i}} \end{array}\right)∼N_{8}\left(\left(\begin{array}{c} 0\\ 0\\ 0\\ 0\\ 0\\ 0\\ 0\\ 0 \end{array}\right),D=\left(\begin{array}{cccccccc} d_{ss} & d_{st} & d_{sa} & d_{sb} & d_{scs} & d_{sct} & d_{sds} & d_{sdt}\\ & d_{tt} & d_{ta} & d_{tb} & d_{tcs} & d_{tct} & d_{tds} & d_{tdt}\\ & & d_{aa} & d_{ab} & d_{acs} & d_{act} & d_{ads} & d_{adt}\\ & & & d_{bb} & d_{bcs} & d_{bct} & d_{bds} & d_{bdt}\\ & & & & d_{cs} & d_{csdt} & d_{csds} & d_{csdt}\\ & & & & & d_{ct} & d_{ctds} & d_{ctdt}\\ & & & & & & d_{ds} & d_{dsdt}\\ & & & & & & & d_{dt} \end{array}\right)\right)$$

Now, we can choose a level x for the covariaite K and calculate the causal effect of a treatment Z on the surrogate marker and outcome measure among subjects with X=x in the ith trial as

$$\Delta_{S_{i}}(x)=E(S_{ij}(1|X_{ij}=x)-S_{ij}(0|X_{ij}=x))\;\;=E(S_{ij}|Z_{ij}=1,X_{ij}=x)-E(S_{ij}|Z_{ij}=0,X_{ij}=x)\;\;=\alpha_{S}+\beta_{S}+\gamma_{S}x+\delta_{S}x+a_{S_{i}}+b_{S_{i}}+c_{S_{i}}x+d_{S_{i}}x-(\alpha_{S}+\gamma_{S}x+a_{S_{i}}+c_{S_{i}}x)\;\;=\beta_{S}+\delta_{S}x+b_{S_{i}}+d_{S_{i}}x$$

Similarly, the causal effect of a treatment Z on the surrogate marker and outcome measure among subjects with X=x in the ith trial is

$$\Delta_{T_{i}}(x)=E(T_{ij}(1|X_{ij}=x)-T_{ij}(0|X_{ij}=x))\;\;=E(T_{ij}|Z_{ij}=1,X_{ij}=x)-E(T_{ij}|Z_{ij}=0,X_{ij}=x)\;\;=\alpha_{T}+\beta_{T}+\gamma_{T}x+\delta_{T}x+a_{T_{i}}+b_{T_{i}}+c_{T_{i}}x+d_{T_{i}}x-(\alpha_{T}+\gamma_{T}x+a_{T_{i}}+c_{T_{i}}x)\;\;=\beta_{T}+\delta_{T}x+b_{T_{i}}+d_{T_{i}}x$$

Now, we can calculate the joint distribution of $\Delta_{S_{i}}(x)$ and $\Delta_{T_{i}}(x)$:

$$E(\Delta_{S_{i}}(x))=E(\beta_{S}+\delta_{S}x+b_{S_{i}}+d_{S_{i}}x)=\beta_{S}+\delta_{S}\;x\;E(\Delta_{T_{i}}(x))=E(\beta_{T}+\delta_{T}x+b_{T_{i}}+d_{T_{i}}x)=\beta_{T}+\delta_{T}x$$

$$Var(\Delta_{S_{i}}(x))=Var(\beta_{S}+\delta_{S}x+b_{S_{i}}+d_{S_{i}}x)=d_{a}a+x^{2}d_{ds}+2xd_{ads}\;Var(\Delta_{T_{i}}(x))=Var(\beta_{T}+\delta_{T}x+b_{T_{i}}+d_{T_{i}}x)=d_{b}b+x^{2}d_{dt}+2xd_{bdt}$$

$$Cov(\Delta_{S_{i}}(x),\Delta_{T_{i}}(x))=Cov(\beta_{S}+\delta_{S}x+b_{S_{i}}+d_{S_{i}}x,\beta_{T}+\delta_{T}x+b_{T_{i}}+d_{T_{i}}x)\;\;=Cov(b_{S_{i}}+d_{S_{i}}x,b_{T_{i}}+d_{T_{i}}x)\;\;=E(b_{S_{i}}b_{T_{i}}+b_{S_{i}}d_{T_{i}}x+b_{T_{i}}d_{S_{i}}x+d_{S_{i}}d_{T_{i}}x^{2})\;\;=Cov(b_{S_{i}},b_{T_{i}})+xCov(b_{S_{i}},d_{T_{i}})+xCov(b_{T_{i}},d_{S_{i}})+x^{2}Cov(d_{S_{i}},d_{T_{i}})\;\;=d_{ab}+xd_{adt}+xd_{bds}+x^{2}d_{dsdt}$$

Thus, $\Delta_{S_{i}}$ and $\Delta_{T_{i}}$ have the joint distribution:

$$\left(\begin{array}{c} \Delta_{S_{i}}\\ \Delta_{T_{i}} \end{array}\right)∼N_{2}\left(\left(\begin{array}{c} \beta_{S}+\delta_{S}\;x\\ \beta_{T}+\delta_{T}\;x \end{array}\right),D^{*}=\left(\begin{array}{cc} d_{aa}^{*} & d_{ab}^{*}\\ & d_{bb}^{*} \end{array}\right)\right)$$

where

$$d_{aa}^{*}=d_{aa}+x^{2}d_{ds}+2xd_{ads}\;d_{ab}^{*}=d_{ab}+xd_{adt}+xd_{bds}+x^{2}d_{dsdt}\;d_{bb}^{*}=d_{bb}+x^{2}d_{dt}+2xd_{bdt}$$

**Figure 3.**
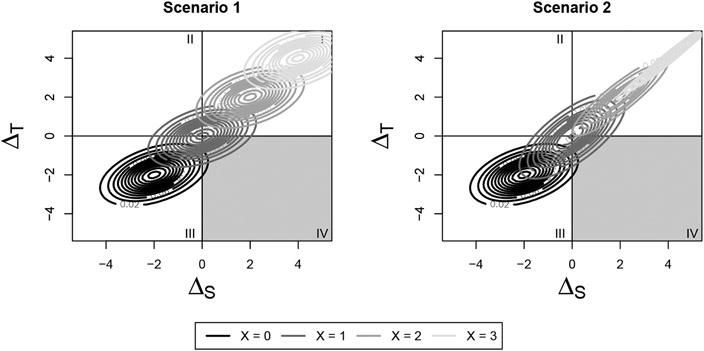
Changes to the joint distribution of $\Delta_{S}$ and $\Delta_{T}$ dependent on a continuous covariate X: (1) Scenario 1: The effects of a continuous covariate X on surrogate and outcome is constant across trials, resulting in a mean shift of the overall distribution based on the value of X. (2) Scenario 2: The effects of a continuous covariate X on surrogate and outcome differs across trials, resulting in both a mean shift and variance change for different values of X.

This distribution consists of both a mean shift and change in variance compared with the original, no-subgroup distribution. To visualize this, refer to Scenario 2 in Figures 2 and 3. The change in risk occurs from both a mean shift and change in variance of the overall joint distribution by covariate level. We can use this distribution to construct the four surrogate paradox measures proposed by Elliott et al.

#### 3.2.1. Scenario 2: $\Psi_{SP13}(x)$

Using the new joint distribution, the probability that the N+1th trial will yield treatment effects on the marker and outcome in the same direction is given by

$$\Psi_{SP13}(x)=1-\Phi_{1}(0;\beta_{S}+\delta_{S}x,d_{aa}^{*})-\Phi_{1}(0,\beta_{T}+\delta_{T}x,d_{bb}^{*})+2\Phi_{2}\left(\left(\begin{array}{c} 0\\ 0 \end{array}\right),\left(\begin{array}{c} \beta_{S}+\delta_{S}\;x\\ \beta_{T}+\delta_{T}\;x \end{array}\right),\left(\begin{array}{cc} d_{aa}^{*} & d_{ab}^{*}\\ & d_{bb}^{*} \end{array}\right)\right)$$

where $\Phi_{k}(x;\Theta ,\Psi )$ is the cumulative distribution function of a k-variate normal distribution with mean Θ and variance Ψ.

#### 3.2.2. Scenario 2: $\Psi_{SP123}(x)$

Under the new joint distribution, the probability that the treatment effects for the outcome will be harmful given that the treatment effect on the marker is beneficial is given by

$$\Psi_{SP123}(x)=1-\Phi_{1}(0;\beta_{T}+\delta_{T}x,d_{bb}^{*})+\Phi_{2}\left(\left(\begin{array}{c} 0\\ 0 \end{array}\right),\left(\begin{array}{c} \beta_{S}+\delta_{S}\;x\\ \beta_{T}+\delta_{T}\;x \end{array}\right),\left(\begin{array}{cc} d_{aa}^{*} & d_{ab}^{*}\\ & d_{bb}^{*} \end{array}\right)\right)$$

#### 3.2.3. Scenario 2: ${\hat{\Psi }}_{PS13_{N}}(x)$

Let ${ϒ}_{ij}=\left(\begin{array}{c} S_{ij}\\ T_{ij} \end{array}\right)$ constitute the surrogate marker and outcome for each subject,

$$M_{ij}=\left(\begin{array}{cccccccc} 1 & 0 & Z_{ij} & 0 & x & 0 & xZ_{ij} & 0\\ 0 & 1 & 0 & Z_{ij} & 0 & x & 0 & xZ_{ij} \end{array}\right)$$

be the fixed effect matrix associated with the parameters $\mu =(\alpha_{S},\alpha_{T},\beta_{S},\beta_{T},\gamma_{S},\gamma_{T},\delta_{S},\delta_{T}{)}^{T}$, and let

$$W_{ij}=\left(\begin{array}{cccccccc} 1 & 0 & Z_{ij} & 0 & x & 0 & xZ_{ij} & 0\\ 0 & 1 & 0 & Z_{ij} & 0 & x & 0 & xZ_{ij} \end{array}\right)$$

be the random effect matrix associated with $\gamma_{i}=(a_{S_{i}},a_{T_{i}},b_{S_{i}},b_{T_{i}},c_{S_{i}},c_{T_{i}},d_{S_{i}},d_{T_{N}}{)}^{T}$. Let $M_{i}$, ${ϒ}_{i}$, and $W_{i}$ represent the stacked elements of $M_{ij}$, ${ϒ}_{ij}$, and $W_{ij}$.

Consider the vector of random effects $\gamma_{N}=(a_{S_{N}},a_{T_{N}},b_{S_{N}},b_{T_{N}},c_{S_{N}},c_{T_{N}},d_{S_{N}},d_{T_{N}}{)}^{T}$, then the conditional distribution of $\gamma_{N}|{ϒ}_{N}$ can be found by considering the joint distribution of $\gamma_{N}∼MVN(X_{N}\mu ,V_{N})$ and $\gamma_{N}∼MVN(0,D^{*})$ and $cov({ϒ}_{N},\gamma_{N})=W_{N}D^{*}$ for $V_{N}=W_{N}D^{*}W_{N}^{T}+R$, with R representing a $2n_{N}+2n_{N}$ matrix with block diagonals of σ as before.

$$\gamma_{N}|{ϒ}_{N}∼MVN({\tilde{\gamma }}_{N},{\tilde{D}}_{N})$$

where ${\tilde{\gamma }}_{N}\;=\;D^{*}W_{N}^{T}V_{N}^{-1}({ϒ}_{N}-X_{N}\mu )$ and ${\tilde{D}}_{N}\;=\;D^{*}-D^{*}W_{N}^{T}V_{N}^{-1}W_{N}D^{*}$. From here, the measure of surrogate paradox risk is given by

$${\hat{\Psi }}_{SP13_{N}}(x)=1-\Phi_{1}(0;{\hat{\tilde{\beta }}}_{S_{N}}+{\hat{\tilde{\delta }}}_{S_{N}}x,{\hat{\tilde{d}}}_{33N})-\Phi_{1}(0;{\hat{\tilde{\beta }}}_{T_{N}}+{\hat{\tilde{\delta }}}_{T_{N}}x,{\hat{\tilde{d}}}_{44_{N}})\;\;+2\Phi_{2}\left(\left(\begin{array}{c} 0\\ 0 \end{array}\right),\left(\begin{array}{c} {\hat{\tilde{\beta }}}_{S_{N}}+{\hat{\tilde{\delta }}}_{S_{N}}x\\ {\hat{\tilde{\beta }}}_{T_{N}}+{\hat{\tilde{\delta }}}_{T_{N}}x \end{array}\right),\left(\begin{array}{cc} {\hat{\tilde{d}}}_{33_{N}} & {\hat{\tilde{d}}}_{34_{N}}\\ & {\hat{\tilde{d}}}_{44_{N}} \end{array}\right)\right)$$

where ${\hat{\tilde{\beta }}}_{S_{N}}={\hat{\beta }}_{S}+{\hat{\delta }}_{S}+{\hat{\tilde{b}}}_{S_{N}}$ for ${\hat{\beta }}_{S}$ and ${\hat{\delta }}_{S}$ corresponding to the third and seventh elements of the estimate of μ and ${\hat{\tilde{b}}}_{S_{N}}$ corresponding to the third element of the estimate of ${\tilde{\gamma }}_{N}$, ${\hat{\tilde{\beta }}}_{T_{N}}={\hat{\beta }}_{T}+{\hat{\delta }}_{T}+{\hat{\tilde{b}}}_{T_{N}}$ for ${\hat{\beta }}_{T}$ and ${\hat{\delta }}_{T}$ corresponding to the fourth and eighth element of the estimate of μ and ${\hat{\tilde{b}}}_{T_{N}}$ corresponding to the fourth element of the estimate of ${\tilde{\gamma }}_{N}$, ${\hat{\tilde{d}}}_{kl_{N}}$ corresponding to the k, l element of the estimate of ${\tilde{D}}_{N}$. Similarly, we can derive ${\hat{\Psi }}_{SP123_{N}}(x)$.

#### 3.2.4. Scenario 2: s Value

In the fourth surrogate paradox measure, Elliott et al. consider the minimum observed beneficial treatment effect for a marker that can reduce the probability that the true treatment effect for the outcome is harmful. When considering covariate subgroups, we can compute $O_{S_{i}}$ for each covariate level and call it $O_{S_{i}}(x)$:

$$O_{S_{i}}(x)=\frac{\sum_{j:X_{ij}=x}Z_{ij}S_{ij}}{\sum_{j:X_{ij}=x}Z_{ij}}-\frac{\sum_{j:X_{ij}=x}(1-Z_{ij})S_{ij}}{\sum_{j:X_{ij}=x}(1-Z_{ij})}$$

$O_{S_{i}}(x)$ represents the difference between the observed surrogate marker means under treatment and control within a fixed level of X. Then, the joint distribution of the true treatment effect on the outcome and the observed treatment effect on the surrogate marker is given by

$$\left(\begin{array}{c} O_{S_{i}}(x)\\ \Delta_{T_{i}}(x) \end{array}\right)∼N_{2}\left(\left(\begin{array}{c} \beta_{S}+\delta_{S}\;x\\ \beta_{T}+\delta_{T}\;x \end{array}\right),\left(\begin{array}{cc} {\tilde{d}}_{aa}^{*} & d_{ab}^{*}\\ & d_{bb}^{*} \end{array}\right)\right)$$

where ${\tilde{d}}_{aa}^{*}=d_{aa}^{*}+\sigma_{ss}(1∕n_{1ix}+1∕n_{0ix})$, $n_{1ix}=\sum_{j:X_{ij}=x}Z_{ij}$, and $n_{0ix}=\sum_{j:X_{ij}=x}(1-Z_{ij})$. So, the distribution of the true treatment effect on the outcome $\Delta_{Ti}(x)$ conditional on a given observed treatment effect $O_{Si}(x)$ within the group having X=x is

$$\Delta_{Ti}|O_{Si}(x)=s∼N(\beta_{T}+\delta_{T}x+d_{ab}^{*}∕{\tilde{d}}_{aa}^{*}(s-(\beta_{S}+\delta_{S}x)),d_{bb}^{*}-(d_{ab}^{*}{)}^{2}∕{\tilde{d}}_{aa}^{*})$$

and

$$P(\Delta_{Ti}<0|O_{Si}=s,X=x)=\Phi \left(\frac{-(\beta_{T}+\delta_{T}x+d_{ab}^{*}∕{\tilde{d}}_{aa}^{*}(s-(\beta_{S}+\delta_{S}x)))}{\sqrt{d_{bb}^{*}-(d_{ab}^{*}{)}^{2}∕{\tilde{d}}_{aa}^{*}}};0,1\right)$$

The value of s that will ensure that the probability that $\Delta_{Ti}(x)$ is negative is less than or equal to a preset level α:

$$s\geq \beta_{S}+\delta_{S}x-\frac{{\tilde{d}}_{aa}^{*}}{d_{ab}^{*}}\left(\Phi (\alpha ;0,1{)}^{-1}\sqrt{\frac{{\tilde{d}}_{aa}^{*}d_{bb}^{*}-(d_{ab}^{*}{)}^{2}}{{\tilde{d}}_{aa}^{*}}}+\beta_{T}+\delta_{T}x\right)$$

## 4. Bayesian Estimation

In this section, we describe how to obtain estimates and inferences for the proposed measures using a Bayesian frameworks for scenario 2, which is a generalization of scenario 1 that allows for covariate effects and interactions to differ by study. It is also possible to estimate the measures using a maximum likelihood (ML) or reduced maximum likelihood (REML) approach, although it is often not computationally feasible in practice without large sample sizes, so we focused on a Bayesian estimation approach in this paper. Details of the ML/REML estimation approach are provided in the Appendix A.

The estimation can also be conducted using a fully Bayesian approach, with priors placed on μ, D, and σ. We obtain draws of the parameters from a Markov chain Monte Carlo and transform them to obtain $p(\psi_{SP13}|ϒ)$ and $p(\psi_{SP123}|ϒ)$, the posterior distributions of $\psi_{SP13}$ and $\psi_{SP123}$. We place a multivariate normal prior on the fixed effects, $\mu =(\alpha_{S},\alpha_{T},\beta_{S},\beta_{T},\gamma_{S},\gamma_{T},\delta_{S},\delta_{T}{)}^{T}$, such that $\mu ∼N_{8}(0,\Sigma_{0})$. We place Wishart priors on the variance parameters D and σ such that $\sigma^{-1}W(v_{\sigma },G)$ and $D^{-1}W(v_{D},F)$. Then, we can obtain the conditional posterior distributions for each of the parameters of interest as

$$D^{-1}|\cdot ∼W\left(N+v_{D},{\left(\Sigma_{i=1}^{N}\gamma_{i}\gamma_{i}^{T}+F^{-1}\right)}^{-1}\right)\;\sigma^{-1}|\cdot ∼W\left(\Sigma_{i=1}^{N}n_{i}+v_{\sigma },(S_{1}+G^{-1}{)}^{-1}\right)\;\gamma_{i}^{-1}|\cdot ∼N_{8}\left(S_{2}\left(\Sigma_{j=1}^{n_{i}}M_{ij}^{T}\sigma^{-1}({ϒ}_{ij}-M_{ij}\mu )\right),S_{2}\right)\;\mu^{-1}|\cdot ∼N_{8}\left(S_{3}\left(\Sigma_{i=1}^{N}\Sigma_{j=1}^{n_{i}}M_{ij}^{T}\sigma^{-1}({ϒ}_{ij}-W_{ij}\gamma_{i})\right),S_{3}\right)$$

with

$$S_{1}=\Sigma_{i=1}^{N}\Sigma_{j=1}^{n_{i}}({ϒ}_{ij}-M_{ij}\mu -W_{ij}\gamma_{i})({ϒ}_{ij}-M_{ij}\mu -W_{ij}\gamma_{i}{)}^{T}\;S_{2}={\left(\Sigma_{j=1}^{n_{i}}M_{ij}^{T}\sigma^{-1}M_{ij}+D^{-1}\right)}^{-1}\;S_{3}={\left(\Sigma_{i=1}^{N}\Sigma_{j=1}^{n_{i}}M_{ij}^{T}\sigma^{-1}M_{ij}+\Sigma_{0}^{-1}\right)}^{-1}$$

Using the conditional posterior distributions and a Gibbs sampling routine, we can obtain draws from the posterior distributions of each of the parameters of interest.

## 5. Testing

In order to determine which scenario is the best fit for a particular analysis, we would need some intuition as to whether the effect of a covariate X on the outcome differs based on the study and whether that effect also differs based on treatment. If there is no intuition as to whether the covariate effect differs by center, it may be of interest to test which scenario is the most appropriate for the observed meta-analytic data. This amounts to jointly testing the null hypotheses that all of the variances and covariances associated with the covariate random effects are equal to zero.


$$D=\left(\begin{array}{cccccccc} d_{ss} & d_{st} & d_{sa} & d_{sb} & 0 & 0 & 0 & 0\\ & d_{tt} & d_{ta} & d_{tb} & 0 & 0 & 0 & 0\\ & & d_{aa} & d_{ab} & 0 & 0 & 0 & 0\\ & & & d_{bb} & 0 & 0 & 0 & 0\\ & & & & 0 & 0 & 0 & 0\\ & & & & & 0 & 0 & 0\\ & & & & & & 0 & 0\\ & & & & & & & 0 \end{array}\right)$$

Since variances are positive, testing whether they are equal to zero means we are testing a null hypothesis on the boundary of the parameter space, and the usual chi-square distribution of the likelihood ratio statistics under this null hypothesis is incorrect. Drikvandi et al. propose a test statistic based on the variance least square estimator of variance components, as well as a permutation test to approximate its finite sample distribution [12]. Under the Bayesian framework, Ariyo et al. recommend using the marginal deviance information criterion (DIC) or the marginal widely applicable information criterion (WAIC) to evaluate the need for random effects [13] by comparing the criterion value between the model including the random effects and a model excluding all the covariate-related random effects.

## 6. Simulations

We perform simulations under several surrogacy scenarios to examine the properties of the proposed estimators as a function of a binary covariate X. We generate data under scenario 1 (the effect of X on the surrogate and outcome is constant across trials) and scenario 2 (the effect of X on the surrogate and outcome is not constant across trials). For scenario 1, we generate data assuming $\alpha_{S}=\alpha_{T}=1$, $\beta_{S}=2,$, $\beta_{T}=1$, $\gamma_{S}=\gamma_{T}=0$, and $\delta_{S}=-1$, $\delta_{T}=1$. For the variance components, we assume $d_{ss}=d_{tt}=d_{aa}=d_{bb}=1$, $d_{ab}=0.5$, and $d_{st}=d_{sa}=d_{sb}=d_{ta}=d_{tb}=d_{ab}=0.3$. For scenario 2, we generate data using the same parameters as scenario 1 and assume the new variance components $d_{cs}=d_{ct}=d_{ds}=d_{dt}=1$ and that all the new off-diagonal components $d_{scs}-d_{dsdt}$ are set to 0.3. Under each scenario, we simulate 200 studies with 30 or 100 clusters, each of size 20, 50, or 500, representing 30 or 100 repeated trials of the same treatment, surrogate, and true endpoint combination, each with either 20, 50, or 500 participants. Half of the participants in each trial are randomly assigned to either placebo or control.

We used a Gibbs sampling routine, as described in Section 4, with a multivariate normal prior for the fixed effects, such that $\left(\alpha_{S},\alpha_{T},\beta_{S},\beta_{T},\gamma_{S},\gamma_{T},\delta_{S},\delta_{T})∼N_{8}(0,10^{6}I_{8}\right)$, and Wishart priors for the inverse of the covariance matrices of the form $W(q+1,(1∕(q+2))∕I_{q})$, where q is the length of the associated vector of covariance effects. We sample from the derived conditional posterior distributions to obtain draws of the proposed estimators. Tables 1 and 2 contain the point estimates, standard errors, bias, and coverage rates for $\psi_{SP13}(X)$, $\psi_{SP123}(x)$, and s, with 30 and 100 trials, respectively. The true value of s assumes that there is equal distribution of subjects between each of the treatment and covariate categories. To estimate $\psi_{SP13_{N}}$, we considered the final study to have only half of the data of the other trials. Although it is also possible to conduct this analysis with a ML/REML estimation approach, as described in the Appendix A, we ran into computation issues when estimating the large number of random effects using reasonable sample sizes and have therefore presented only the simulation results for the Bayesian approach.

We observed some minimal bias in estimating $\psi_{SP13}$, $\psi_{SP123}$, and $\psi_{SP13_{N}}$ with either 30 or 100 trials, each of size 20, 50, or 500 subjects. However, with the estimate of s, we found that the lower number of trials and lower number of subjects resulted in unstable estimates with very large bias and variance. The observed coverage rates of the credible intervals were below the nominal level for some estimates of $\psi_{SP13}$ and $\Psi_{SP123}$ in both scenarios, demonstrating the need for large numbers of trials and subjects per trial when there is a desire to identify the risk of surrogate paradox in subpopulations.

As a sensitivity analysis, we also considered two simulation settings with data that were not normally distributed to assess the robustness of our proposed method to model misspecification. We generated data using a T Distribution with 15 degrees of freedom, as well as a skew normal distribution with α equal to 0.1 times the location and scale parameters and centered at 0. The data generated under the T distribution allow us to assess whether the method is robust to a situation in which the normality assumption is violated in the tails of the distribution [14]. The data generated under the skew normal distribution consider a situation in which the data are distributed asymmetrically, as carried out in prior similar sensitivity analyses [15]. For each sensitivity analysis, we generated 30 trials, each with 50 subjects, and considered the bias, standard error, and coverage of $\psi_{SP13}$ and $\psi_{SP123}$. The true value of each of the parameters of interest was estimated empirically by taking one million draws of $\Delta_{S}$ and $\Delta_{T}$ and computing $\psi_{SP13}$ and $\psi_{SP123}$ from the proportion of draws that fell into each of the relevant quadrants. The results of the sensitivity analysis are shown in Table 3. Under these deviations from normality, we had small increases in bias and standard error but still maintained high coverage rates. As the number of required parameters increased in scenario 2, the coverage rates also decreased, as we would expect.

## 7. Applications

### 7.1. Collaborative Initial Glaucoma Treatment Study

We apply the proposed method to data from the Collaborative Initial Glaucoma Treatment Study (CIGTS) [16]. The CIGTS trial was a multicenter randomized clinical trial that contrasted initial surgical therapy versus initial medical therapy to treat glaucoma, with reduction in intraocular pressure (IOP) as one of its outcome measures. A total of 607 patients were enrolled in the study, and 307 were randomized to the drug arm. IOP was recorded in mmHg at baseline, 3 months, 6 months, and every 6 months thereafter. We consider the measurement of IOP at 18 months after beginning treatment as a surrogate for the true endpoint of interest: IOP at 96 months. We consider the 14 centers at which the study was conducted to be the trial-level replicates. Missing data were imputed using single imputation with a linear mixed model with a random effect for trial, a quadratic trend for time, an effect for treatment, and an interaction between time and treatment. The estimates of the between-trial covariance matrix, D, are not positive definite, so only the results (estimates and 95% credible intervals(CIs)) from the Bayesian estimation procedure are presented. As in the simulation study, we used a Gibbs sampling routine, as described in Section 4, with a multivariate normal prior for the fixed effects, such that $\left(\alpha_{S},\alpha_{T},\beta_{S},\beta_{T},\gamma_{S},\gamma_{T},\delta_{S},\delta_{T})∼N_{8}(0,10^{6}I_{8}\right)$, and Wishart priors for the inverse of the covariance matrices of the form $W(q+1,(1∕(q+2))I_{q})$, where q is the length of the associated vector of covariance effects. The $R_{trial}^{2}$ measure of surrogacy is 0.49, indicating a moderate quality surrogate by the Buyse criteria [6].

In order to illustrate our proposed methods, we consider two covariates: sex (female, male) and age (<60, ≥60), and compute $\Psi_{SP13}$ and $\Psi_{SP123}$ for each variable category under both proposed scenarios. The results are shown in Table 4.

In scenario 1, we exclude all of the random effects for the included covariates. As we can see, overall, there is a small probability of experiencing the surrogate paradox when using early IOP as a surrogate for later IOP in this trial, since the 95% credible intervals of the measures are close to 1. This does not change significantly when comparing the overall $\Psi_{SP13}$ and $\Psi_{SP123}$ with the covariate adjustments, implying that there is no evidence of a significant difference between the risk of surrogate paradox by age or gender. In scenario 2, we estimate all of the random effects for the included covariates, allowing the effect of the covariate and the interaction between the covariate and treatment to differ by study center. In this scenario, we observe some differences between the risk of surrogate paradox by subgroup. Notably, it seems as though males and people aged 60 or over are at a higher risk of experiencing the surrogate paradox in a new trial compared with females and people under the age of 60, respectively. However, the difference in their risk of dangerous surrogate paradox is minimal. In both scenarios, the measure of *s* is too unstable to provide useful inference.

Using WAIC as a model selection tool, we find that there is a WAIC difference of 380 between the models for scenarios 1 and 2 for the model including sex as a covariate, and a WAIC difference of 815 for the model including age as a covariate, and conclude that the models including the additional random effects (scenario 2) are a better fit in this data example. The data for this trial are not publicly available.

### 7.2. Trial of Preventing Hypertension

Our second illustrative example comes from the Trial of Preventing Hypertension (TROPHY) [17]. This multicenter randomized trial compared the effects of two years of treatment with Candesartan versus the standard of care on the incidence of hypertension in patients with prehypertension. Blood pressure and hypertension status were collected at baseline, 1 month and 3 months post randomization, and then every 3 months for a total of two years of follow-up. To illustrate our proposed methods, we consider the average of systolic and diastolic pressure at 1 month as a surrogate for the average of systolic and diastolic pressure at 12 months. Although the primary endpoint of interest in the original trial was a binary indicator of developing hypertension, we used the endpoint of average systolic and diastolic pressure at 12 months, since our method has currently only been developed for normally distributed outcomes. After developing hypertension patients were switched to a new treatment regimen, resulting in some missing data in both the surrogate measured at 1 month and the true endpoint measured at 12 months. These missing data were imputed using a model that was stratified by treatment and gender and included the following baseline covariates: age, race, weight, body mass index, systolic blood pressure, diastolic blood pressure, total cholesterol, high-density lipoprotein cholesterol (HDL), low-density lipoprotein (LDL), HDL:LDL ratio, triglycerides, fasting glucose, total insulin, and creatinine. For missing outcome values at 12 months, the imputation model also included the blood pressure measurements up to the 12th month. We consider the 69 centers at which the study was conducted to be the trial-level replicates. There were a total of 772 patients included in the original analysis. After removing centers with patients in only one treatment arm, there were a remaining 62 centers and 764 patients, 389 of which received the treatment. The size of the remaining centers ranged from 2 patients to 46 patients. When applying the REML estimation method, the covariance matrix was nonpositive-definite (likely due to the small sample size at some centers), so we only present the results (estimates and 95% credible intervals (CIs)) from the Bayesian estimation procedure.

In order to illustrate our proposed methods, we consider two covariates: sex (female, male) and age (<50, ≥50), and compute $\Psi_{SP13}$ and $\Psi_{SP123}$ for each variable category under both proposed scenarios. The results are shown in Table 5.

The results indicate that, overall, there is very little risk of the surrogate paradox when considering the effect Candesartan on the average of systolic and diastolic blood pressure at 1 month as a surrogate for the average of systolic and diastolic blood pressure at 12 months. Although there are minor differences between the risk of surrogate paradox (measured through both $\Psi_{SP13}$ and $\Psi_{SP123}$) by gender and age, the credible intervals overlap between the groups, indicating no significant difference between their risk of surrogate paradox. As in the previous example, the measure of s is too unstable to provide useful inference, consistent with our simulation study that indicated a large number of trials would be required to obtain useful inference for this quantity.

Using WAIC as a model selection tool, we find that there is a WAIC difference of 120 between the models for scenarios 1 and 2 for the model with sex as a covariate, and a WAIC differnce of 83 for the model with age as a covariate, and conclude that the models including the additional random effects (scenario 2) are better fitting in this data example. However, qualitatively, the results between the two scenarios are quite similar, and a simpler model may be preferred. The data for this trial are not publicly available.

## 8. Discussion

Surrogate outcomes are commonly used in clinical trials, and their prevalence has led to the development of innovative trial designs that aim to efficiently use the additional information provided by surrogate outcomes [18-20]. Despite the valuable additional information that surrogate outcomes provide, their use also comes with risk. Evaluating the quality of a chosen surrogate to prevent the surrogate paradox should be an important step in both the design and analysis of clinical trials.

There are several existing approaches for evaluating surrogate outcome efficacy, but some apparently “good” surrogates under these methods may still experience the “surrogate paradox”, in which the treatment has a positive effect on the surrogate endpoint but a negative effect on the true endpoint. The meta-analytic causal association approach to surrogate validation is particularly useful in assessing the risk of surrogate paradox. In this paper, we develop methods to measure the risk of the surrogate paradox in subpopulations when there are data available on multiple trials of similar treatments on the same surrogate and outcome. Using measures of surrogate paradox risk can prevent the occurrence of the surrogate paradox in new trials and protect the health of study participants.

Incorporating covariate information can provide valuable insights into the mechanism of the surrogate paradox and identify groups that are particularly vulnerable to the paradox. This additional information can tell us about the transferability of surrogates from one trial to the next, depending on their study population. It can also help assess the risk of using a proposed surrogate in a new trial depending on the demographic distribution of the new study population. Researchers can incorporate their understanding of whether certain subpopulations are at a higher risk of experiencing the surrogate paradox into the design of new clinical trials of similar treatments that plan to use the same surrogate and true endpoints.

Both our simulations and examples focused on exploring whether the surrogate paradox risk varied with a single scalar covariate. While in principle this could easily be extended to a multiple-covariate setting, in practice, this would typically require a fairly large set number of trials to obtain stable estimates, especially for the “scenario 2” setting, where both the fixed and random effects are associated with multiple covariates. Our simulation study showed that the estimation of some measures can be unstable when there is a small number of trials and subjects. We also considered simulations under mild deviations from normality and were able to retain relatively high coverage rates. The proposed method derives the probabilities of interest assuming normally distributed variables that may not be likely in practice. Future work will consider further violations of the normality assumption, as well as how to account for them when estimating the risk of surrogate paradox.

This work has the potential to be extended to non-normal surrogate and true endpoints. By using a copula model instead of the bivariate normal assumption in this paper, we may be able to consider a larger range of distributions for the surrogate and true endpoints, including binary or time-to-event distributions. We may also be able to consider the situation when the proposed surrogate and true endpoints have differing distributional forms (e.g., an indicator of hypertension as a surrogate for time to cardiac death). Another potential extension is to apply meta-analytic methods to estimate the risk of surrogate paradox when individual-level data on the prior studies are not available. One example would be if we only have the parameter estimates from a series of published papers on the same treatment and endpoint combination and want to use them to estimate the risk of surrogate paradox risk in a newly designed study.

Finally, we note that while we focused on conditional surrogacy paradox estimates—interactions with covariates—this method can also be used to deal with non-normality in the multiple trials setting, with the conditional surrogacy paradox measures averaged to obtain marginal results, using the sample distribution of the covariates to approximate the population density. Thus,

$$\Psi_{SP13}=\int \Psi_{SP13}(x)P(x)dx\approx \frac{1}{n}\sum_{i=1}^{n}{\hat{\Psi }}_{SP13}(x_{i});$$

variance estimates could be obtained by bootstrapping for the REML approaches or via posterior distributions of draws of $\Psi_{SP13}$ obtained by averaging the draws of $\Psi_{SP13}(x_{i})$.

The code for implementing these methods is available at github.com/fatemashafie.

## Figures and Tables

**Table 1.** Simulation Results for 30 trials.

				20 Subjects		50 Subjects		500 Subjects	
	Quantity	X	True Value	Bias (SE)	Coverage	Bias (SE)	Coverage	Bias (SE)	Coverage
Scenario 1	$\psi_{SP13}$	0	0.850	0.04 (0.06)	98%	0.04 (0.05)	94%	0.05 (0.04)	84%
		1	0.845	0.04 (0.06)	98%	0.04 (0.05)	95%	0.04 (0.04)	88%
	$\psi_{SP123}$	0	0.854	0.04 (0.06)	99%	0.04 (0.05)	95%	0.04 (0.04)	86%
		1	0.991	0.01 (0.02)	99%	0.01 (0.01)	96%	0.01 (0.01)	92%
	$\psi_{SP13_{N}}$	0	0.850	0.07 (0.08)	98%	0.06 (0.06)	95%	0.05 (0.05)	87%
		1	0.845	0.08 (0.10)	98%	0.06 (0.07)	96%	0.04 (0.05)	84%
	s	0	−3.56	59.81 (267)	100%	15.96 (671)	100%	5.78 (243)	99%
		1	−6.82	97.27 (4338)	100%	22.12 (929)	99%	7.95 (333)	99%
Scenario 2	$\psi_{SP13}$	0	0.850	0.05 (0.05)	93%	0.05 (0.05)	87%	0.05 (0.04)	84%
		1	0.762	0.04 (0.05)	94%	0.04 (0.05)	93%	0.05 (0.04)	84%
	$\psi_{SP123}$	0	0.855	0.04 (0.05)	96%	0.04 (0.04)	90%	0.04 (0.04)	89%
		1	0.961	0.03 (0.02)	87%	0.02 (0.02)	86%	0.02 (0.02)	85%
	$\psi_{SP13N}$	0	0.850	0. (0.)	99%	0. (0.)	95%	0. (0.)	86%
		1	0.762	0. (0.)	99%	0. (0.)	96%	0. (0.)	92%
	s	0	−3.56	792.20 (3542)	100%	10.59 (426)	100%	8.48 (311)	100%
		1	−7.31	18.10 (736)	100%	10.23 (384)	100%	13.37 (478)	100%

**Table 2.** Simulation Results for 100 trials.

				20 Subjects		50 Subjects		500 Subjects	
	Quantity	X	True Value	Bias (SE)	Coverage	Bias (SE)	Coverage	Bias (SE)	Coverage
Scenario 1	$\psi_{SP13}$	0	0.850	0.02 (0.03)	97%	0.03 (0.03)	93%	0.02 (0.02)	89%
		1	0.845	0.02 (0.03)	95%	0.02 (0.03)	94%	0.02 (0.02)	85%
	$\psi_{SP123}$	0	0.854	0.03 (0.03)	95%	0.02 (0.03)	96%	0.02 (0.02)	90%
		1	0.991	0.003 (0.01)	95%	0.004 (0.01)	96%	0.004 (0.02)	92%
	$\psi_{SP13N}$	0	0.850	0.02 (0.04)	96%	0.03 (0.03)	92%	0.02 (0.02)	89%
		1	0.762	0.02 (0.04)	95%	0.03 (0.03)	94%	0.02 (0.02)	85%
	s	0	−3.27	1.06 (3.98)	92%	0.85 (3.76)	97%	0.77 (2.29)	97%
		1	−6.43	1.44 (5.53)	92%	0.67 (5.16)	97%	0.61 (3.16)	98%
Scenario 2	$\psi_{SP13}$	0	0.850	0.03 (0.03)	89%	0.02 (0.03)	94%	0.03 (0.02)	86%
		1	0.762	0.02 (0.03)	96%	0.02 (0.03)	93%	0.03 (0.02)	85%
	$\psi_{SP123}$	0	0.855	0.03 (0.03)	92%	0.02 (0.03)	95%	0.03 (0.02)	83%
		1	0.961	0.01 (0.01)	87%	0.01 (0.01)	89%	0.01 (0.01)	82%
	$\psi_{SP13N}$	0	0.850	0.06 (0.06)	89%	0.03 (0.05)	93%	0.03 (0.04)	84%
		1	0.762	0.06 (0.08)	93%	0.05 (0.06)	93%	0.03 (0.03)	85%
	s	0	−3.27	2.90 (78.60)	99%	1.93 (7.12)	99%	0.87 (2.99)	97%
		1	−7.13	2.32 (19.42)	93%	2.02 (4.64)	94%	1.04 (2.22)	96%

**Table 3.** Sensitivity to model misspecification: each sensitivity analysis considered 30 simulated trials, each with 50 subjects.

				*T*_15_ Distribution		Skew Normal Distribution	
	Quantity	X	True Value	Bias (SE)	Coverage	Bias (SE)	Coverage
Scenario 1	$\psi_{SP13}$	0	0.845	0.04 (0.05)	96%	0.04 (0.05)	94%
		1	0.846	0.04 (0.05)	94%	0.05 (0.05)	95%
	$\psi_{SP123}$	0	0.854	0.04 (0.05)	97%	0.04 (0.05)	95%
		1	0.990	0.01 (0.01)	98%	0.01 (0.01)	97%
Scenario 2	$\psi_{SP13}$	0	0.845	0.05 (0.05)	89%	0.05 (0.05)	88%
		1	0.762	0.04 (0.04)	88%	0.04 (0.04)	91%
	$\psi_{SP123}$	0	0.854	0.04 (0.04)	92%	0.04 (0.04)	91%
		1	0.961	0.02 (0.02)	87%	0.02 (0.02)	86%

**Table 4.** Results of application to Collaborative Initial Glaucoma Treatment Study dataset.

		Scenario 1						Scenario 2					
	Quantity	$\psi_{\mathrm{SP}13}$	95% CI	$\psi_{\mathrm{SP}123}$	95% CI	s	95% CI	$\psi_{\mathrm{SP}13}$	95% CI	$\psi_{\mathrm{SP}123}$	95% CI	s	95% CI
Sex	Female	0.96	(0.76, >0.99)	0.99	(0.95, >0.99)	−3.6	(−34.7, 31.2)	0.93	(0.71, >0.99)	0.99	(0.93, >0.99)	−4.9	(−46.2, 34.5)
	Male	0.97	(0.79, >0.99)	0.99	(0.91, >0.99)	−3.2	(−30.0, 21.7)	0.86	(0.64, 0.97)	0.96	(0.81, >0.99)	−2.4	(−30.7, 31.2)
Age	<60	0.97	(0.78, >0.99)	0.99	(0.90, >0.99)	−1.7	(−32.2, 23.8)	0.92	(0.71, >0.99)	0.98	(0.83, >0.99)	−2.6	(−27.4, 24.2)
	≥60	0.96	(0.75, >0.99)	0.99	(0.96, >0.99)	−2.8	(−24.3, 23.0)	0.84	(0.61, 0.97)	0.98	(0.87, >0.99)	−1.7	(31.9, 33.6)

**Table 5.** Results of application to Trial of Preventing Hypertension dataset.

		Scenario 1						Scenario 2					
	Quantity	$\psi_{\mathrm{SP}13}$	95% CI	$\psi_{\mathrm{SP}123}$	95% CI	s	95% CI	$\psi_{\mathrm{SP}13}$	95% CI	$\psi_{\mathrm{SP}123}$	95% CI	s	95% CI
Sex	Female	0.99	(0.99, >0.99)	0.99	(0.99, >0.99)	1.0	(−147.2, 138.6)	0.92	(0.89, 0.94)	0.96	(0.93, 0.98)	0.9	(−140.9, 130.1)
	Male	0.99	(0.99, >0.99)	0.99	(0.99, >0.99)	1.4	(−164.8, 154.9)	0.94	(0.92, 0.95)	0.97	(0.95, 0.98)	1.6	(−120.9, 98.1)
Age	<50	0.99	(0.96, >0.99)	0.99	(0.99, >0.99)	−0.9	(−80.7, 74.6)	0.99	(0.96, >0.99)	0.99	(0.99, >0.99)	−0.3	(−143.3, 138.0)
	>50	0.99	(0.99, >0.99)	0.99	(0.99, >0.99)	2.9	(−139.0, 127.7)	0.99	(0.95, >0.99)	0.99	(0.98, >0.99)	1.8	(−132.7, 125.2)

## Data Availability

The data used in this study are not publicly available. Code for implementing the methods is available at github.com/fatemashafie.

## Appendix A. Maximum Likelihood Estimation

To estimate $\psi_{SP13}$, we can use the best linear unbiased estimators from a linear mixed model using either maximum likelihood (ML) or reduced maximum likelihood (REML) estimation. Let ${ϒ}_{ij}=(S_{ij},T_{ij}{)}^{T}$ constitute the surrogate marker and outcome for each subject,

$$M_{ij}=\left(\begin{array}{cccccccc} 1 & 0 & Z_{ij} & 0 & x & 0 & xZ_{ij} & 0\\ 0 & 1 & 0 & Z_{ij} & 0 & x & 0 & xZ_{ij} \end{array}\right)$$

be the fixed effect matrix associated with the parameters $\mu =(\alpha_{S},\alpha_{T},\beta_{S},\beta_{T},\gamma_{S},\gamma_{T},\delta_{S},\delta_{T}{)}^{T}$, and let

$$W_{ij}=\left(\begin{array}{cccccccc} 1 & 0 & Z_{ij} & 0 & x & 0 & xZ_{ij} & 0\\ 0 & 1 & 0 & Z_{ij} & 0 & x & 0 & xZ_{ij} \end{array}\right)$$

be the random effect matrix associated with $\gamma_{i}=(a_{S_{i}},a_{T_{i}},b_{S_{i}},b_{T_{i}},c_{S_{i}},c_{T_{i}},d_{S_{i}},d_{T_{i}}{)}^{T}$. Let $M_{i}$, ${ϒ}_{i}$, and $W_{i}$ represent the stacked elements of $M_{ij}$, ${ϒ}_{ij}$, and $W_{ij}$. Then, consider the model:

$${ϒ}_{i}=M_{i}\mu +W_{i}\gamma_{i}+\epsilon_{i}$$

where $\gamma_{i}∼N_{8}(0,D)$, $\epsilon_{i}∼N_{2n_{i}}(0,\sigma ⊗I_{n_{i}})$, $n_{i}$ is the number of observations in the ith trial, and ⊗ is the Kronecker product operator. Then, ${\hat{\beta }}_{S}$, ${\hat{\beta }}_{T}$, ${\hat{\delta }}_{S}$, and ${\hat{\delta }}_{T}$ are the third, fourth, seventh, and eighth elements of of the ML or REML estimator of μ. Similarly, we can obtain estimates of the needed variance components from the ML or REML estimators of D. Then, we have

$${\hat{\Psi }}_{SP13}(x)=1-\Phi_{1}(0;{\hat{\beta }}_{S}+{\hat{\delta }}_{S}x,{\hat{d}}_{aa}^{*})-\Phi_{1}(0;{\hat{\beta }}_{T}+{\hat{\delta }}_{T}x,{\hat{d}}_{bb}^{*})+2\Phi_{2}\left(\left(\begin{array}{c} 0\\ 0 \end{array}\right),\left(\begin{array}{c} {\hat{\beta }}_{S}+{\hat{\delta }}_{S}\;x\\ {\hat{\beta }}_{T}+{\hat{\delta }}_{T}\;x \end{array}\right),\left(\begin{array}{cc} {\hat{d}}_{aa}^{*} & {\hat{d}}_{ab}^{*}\\ & {\hat{d}}_{bb}^{*} \end{array}\right)\right)$$

where

$${\hat{d}}_{aa}^{*}={\hat{d}}_{aa}+x^{2}{\hat{d}}_{ds}+2x{\hat{d}}_{ads}\;{\hat{d}}_{ab}^{*}={\hat{d}}_{ab}+x{\hat{d}}_{adt}+x{\hat{d}}_{bds}+x^{2}{\hat{d}}_{dsdt}\;{\hat{d}}_{bb}^{*}={\hat{d}}_{bb}+x^{2}{\hat{d}}_{dt}+2x{\hat{d}}_{bdt}$$

Similarly, we can estimate ${\hat{\psi }}_{SP123}$.
